# Short-term safety and tolerability profile of 5-methoxy-N,N-dimethyltryptamine in human subjects: a systematic review of clinical trials

**DOI:** 10.3389/fpsyt.2024.1477996

**Published:** 2024-09-19

**Authors:** Aleksander Kwaśny, Alina Wilkowska, Wiesław Jerzy Cubała

**Affiliations:** Department of Psychiatry, Faculty of Medicine, Medical University of Gdańsk, Gdańsk, Poland

**Keywords:** 5-methoxy-N,N-dimethyltryptamine, 5-MeO-DMT, safety, tolerability, adverse events, side effects

## Abstract

**Introduction:**

Psychedelic agents have regained the attention of pharmaceutical companies as promising treatments for depressive episodes. 5-Methoxy-N,N-dimethyltryptamine (5-MeO-DMT), an atypical psychedelic, is emerging as a potentially effective, novel rapid-acting antidepressant. In this systematic review, we analyze the safety and tolerability evidence from clinical trials.

**Methods:**

Following the Preferred Reporting Items for Systematic Reviews and Meta-analyses (PRISMA) guidelines, electronic databases (PubMed, SCOPUS, Web of Science, EMBASE, and EBSCO) were searched from inception until 15 May 2024 to identify clinical trials (regardless of phase) reporting on short-term safety and tolerability profile of 5-MeO-DMT using the following keywords in various combinations: 5-methoxy-N, N-dimethyltryptamine, 5-MeO-DMT, safety, adverse, adverse reaction, side effects, tolerability, dropout, healthy volunteer, healthy participant, depression, major depressive disorder. Only studies written in English were considered.

**Results:**

Initial search yielded 100 records, out of which 3 met the inclusion criteria. These studies reported on the results from clinical trial phases I and I/II, with a total of 78 participants included; two studies involved healthy volunteers, and one included patients with treatment-resistant depression. Although the data is limited, it confirms a good short-term safety and tolerability profile for 5-MeO-DMT, with no serious adverse events (SAEs) reported. Furthermore, no drop-outs were reported.

**Conclusion:**

5-MeO-DMT administration in human subjects presents favorable short-term safety and tolerability profile. Importantly, no SAEs have been documented, and no adverse events led to participant withdrawal from the studies There is a need for future randomized, double-blind, placebo-controlled trials with larger samples and follow-up to assess potential chronic adverse events.

## 1 Introduction

After the initial scientific interest in psychedelics was curtailed by their classification in Schedule I of the United Nations Convention on Psychotropic Substances in 1971, we are now witnessing a second wave of research (1). This resurgence is a continuation in the development of rapid-acting antidepressants and offers hope for another breakthrough in psychiatry, similar to that of ketamine. Classic serotonin agonists like psilocybin, lysergic acid diethylamide (LSD), and N,N-Dimethyltryptamine (DMT) act primarily as agonists or partial agonists at the serotonin 2A (5-HT2A) receptor (1). 5-MeO-DMT differs from classical psychedelics in terms of receptor selectivity which is why it is referred to as an atypical psychedelic (and sometimes referred to as entheogen) (2, 3) as its 5-HT1A agonism appears to predominate over 5HT2A agonism (4). Halberstadt et al. (5) found in a rodent study that the selectivity for the 5-HT1A receptor is 300-fold greater than for the 5-HT2A receptor.

5-MeO-DMT, a naturally occurring tryptamine with psychedelic properties, is found in various plants, fungi, and animals, and has been used traditionally for thousands of years (2). Limited research on the naturalistic use of 5-MeO-DMT (6), as well as recent human clinical trials (7), suggest it may be an effective and rapidly acting antidepressant. It is orally inactive due to rapid metabolism by monoamine oxidase and the cytochrome P450 2D6 (CYP2D6) enzyme, which converts it into the active metabolite bufotenine (8). Therefore, the most common administration routes are smoking or vapor inhalation, with less common methods including intravenous, intramuscular, rectal, sublingual, or intranasal administration (9). Observational studies have shown that inhalation of 5-MeO-DMT vapor results in a very rapid onset of subjective effects occurring within seconds (10) and lasting up to 20 minutes (11). Conversely, intramuscular injection has a slower onset of subjective effects, beginning between 1 and 6 min after injection, and lasting up to 60 min (10). The rapid onset of action and the short duration of effects of 5-MeO-DMT, compared to classic psychedelics, make it feasible to integrate into clinical trial protocols and allow for conclusive interpretation of the experience. The experiences after 5-MeO-DMT use are described as whiteouts “beyond ordinary human comprehension” often with the amnesia of the experience (2, 12). The quality and intensity of peak experiences may be linked to their long-term antidepressant efficacy (6, 13) Such intensive experiences should not be classified as adverse events; thus, a new definition is required. Consequently, discussing adequate safety reporting is crucial for psychedelics, as traditional methods of data interpretation and acquisition used for conventional antidepressants and dissociatives may not apply to psychedelics.

Considering the urgent need for new treatment strategies for depression, research on 5-MeO-DMT offers new hope for patients. However, the safety and tolerability of this novel agent must be reported meticulously, given its early stage of development. It is also important to investigate various doses and methods of administration in this context.

This systematic review presents safety and tolerability data from clinical trials using 5-MeO-DMT in healthy volunteers and patients with treatment-resistant depression (TRD).

## 2 Methods

### 2.1 Search strategy

Following the guidelines of the Preferred Reporting Items for Systematic Reviews and Meta-analyses (PRISMA) (14), we systematically searched the PubMed, SCOPUS, Web of Science, EMBASE, and EBSCO databases from their inception until 15 May 2024. The protocol for this review was not registered.

The following search strategy was employed: (5-methoxy-N, N-dimethyltryptamine OR 5-MeO-DMT) AND (safety OR adverse events OR adverse reactions OR side effects OR tolerability OR dropout) AND (healthy volunteer OR healthy participant OR depression OR major depressive disorder).

### 2.2 Study eligibility criteria

Studies were included in the systematic review if they met the following criteria: 1) clinical trials regardless of trial phase; 2) involved registries on safety and tolerability; 3) concerned human subjects with or without mood disorders. Only studies written in English were considered for inclusion.

Papers were excluded according to the following criteria: 1) non-refereed abstracts, preprints, letters to the editor, reviews, and systematic reviews; 2) non-clinical setting; 3) naturalistic protocols; 4) animal studies.

### 2.3 Data extraction

Two reviewers (AK, AW) independently conducted database searches and extracted the following data: 1) author and year of publication; 2) study design; 3) number and diagnosis of participants; 4) intervention; 5) inclusion criteria; and 6) safety and tolerability outcomes; 7) dropout rate; 8) follow-up duration. Any discrepancies were resolved through discussion. If consensus was not reached, a third reviewer (WJC) was consulted.

### 2.4 Risk of bias assessment

The evaluation of bias risk in randomized trials was conducted utilizing the methodology endorsed by the Cochrane System Reviewer Manual 5.1, specifically the revised tool for assessing bias in randomized trials known as RoB 2. This assessment encompassed aspects such as sequence generation, allocation concealment, blinding, missing outcome data, selective reporting, and other plausible biases. Subsequently, the identified bias risk was classified into three categories: “low,” “some concerns,” or “high,” as per the guidelines established by Higgins et al. (15) and Sterne et al. (16).

In non-randomized studies involving interventions, bias risk was assessed utilizing the Newcastle-Ottawa Scale (17). This scale allocates a maximum of nine points based on criteria including selection (four stars), comparability (two stars), and outcomes (three stars). Studies achieving a score of seven points or higher were categorized as “good quality” studies. Two independent reviewers (AK and AW) conducted the bias risk assessment for each study, resolving any discrepancies through discussion with the guidance of the project supervisor (WJC).

To visually represent the results of randomized trials, the Robvis tool was employed (18).

## 3 Results

### 3.1 Search results

**Figure 1:**
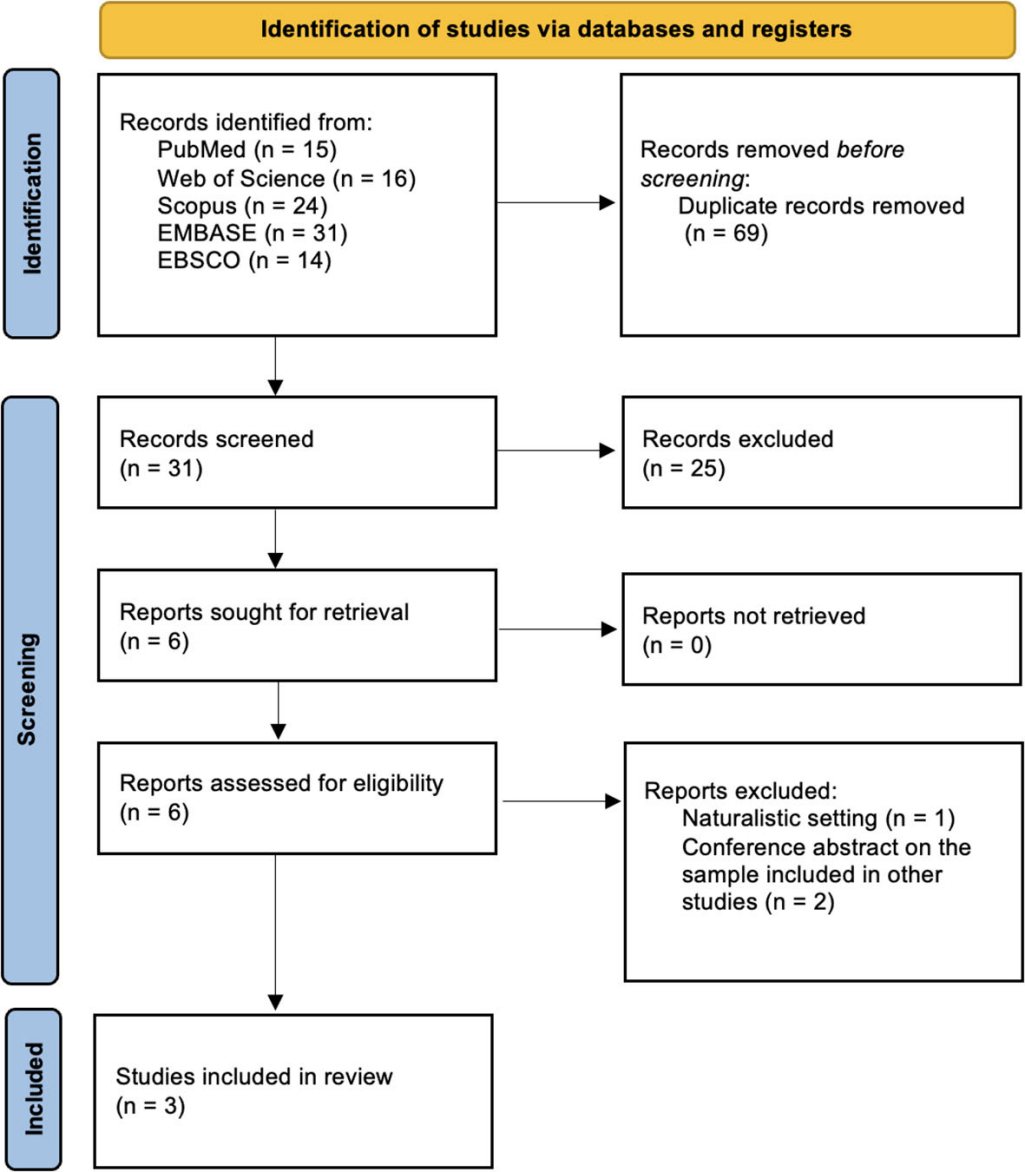
PRISMA flow diagram outlining the study selection process.

The initial search identified 100 records. After removing duplicates (n=69), the remaining records were screened according to the titles, abstracts and keywords. During this process, 25 articles were excluded. The full texts of the 6 remaining records were thoroughly examined in relation to the inclusion and exclusion criteria. However, 3 studies were excluded for the following reasons: 1) naturalistic setting (n=1); 2) conference abstracts on the same sample as included in another paper (n=2). Overall, 3 clinical trials fulfilled the inclusion and exclusion criteria. The study selection process is illustrated in **Figure 1**.

### 3.2 Study characteristics

Out of three included studies, two evaluated 5-MeO-DMT in vaporized formulation, whereas one investigated intranasal administration. Study characteristics are outlined in **Table 1**. Furthermore, the safety profile of both formulations is depicted separately in **Tables 2**, **3**.

**Table 1:** Characteristics of the included studies.

Author	Study design	Participants	Number of participants	Intervention	Formulation	Safety measures	Main findings	Dropout rate	Time of follow-up
Reckweg et al., 2021 (12)	Open-label, single-arm, single-dose	Healthy volunteers with at least two previous experiences with psychedelics, but not within the past 4 weeks.	N = 18	Part A involved single doses (2, 6, 12, 18 mg) Part B: an IDE regimen (6, 12, 18 mg), where up to three doses were administered on a single day	Vaporized	AE reporting Safety laboratory analyses Vital signs measurements ECG monitoring	Vital signs measured at 1 and 3 hours post-administration were not affected. Adverse events were generally mild and resolved spontaneously. The only adverse events of moderate intensity were “increased heart rate” and “fatigue” in one patient. No AEs led to withdrawal.	NS	1 day
Reckweg et al., 2023 (7)	Phase 1: open-label, single-arm, single-dose Phase 2: open-label, single-arm employing IDR	TRD as part of MDD according to DSM-5 for single or recurrent episode	N = 16 Phase 1 N = 8 Phase 2 N = 8	Phase 1: 12 mg or 18 mg single-dose Phase 2: up to three increasing doses (IDR; 6 mg, 12 mg or 18 mg)	Vaporized	AE reporting Safety laboratory analyses Vital signs measurements ECG monitoring	No safety signals were observed regarding SAEs, safety laboratory analyses, vital signs, psychiatric assessments, or cognitive function measures.	0%	7 days
Rucker et al., 2024 (13)	Double-blind, placebo-controlled	Healthy, psychedelic naive participants	N = 44	Participants were divided into seven cohorts. In each cohort participants received a single dose (1, 2.5, 4, 6, 8, 10 or 12 mg) or placebo.	Intranasal	AE reporting Safety laboratory analyses Vital signs measurements ECG monitoring Suicide risk assessment	The most frequently reported AEs were nasal discomfort, nausea, and headache. No clinically significant findings in laboratory parameters, vital signs, or ECGs. A transient increase in heart rate and blood pressure was observed shortly after administration, which resolved within 90 minutes. No participants reported any suicidal thoughts or behaviors, and no symptoms of HPPD or PTSD were evident at follow-up.	0%	7 days

5-MeO-DMT, 5-methoxy-N,N-dimethyltryptamine; ECG, electrocardiogram; HPPD, hallucinogen-persisting perception disorder; IDE, individualized dosing escalation; IDR, individualized dosing regimen; MDD, major depressive disorder; TRD, treatment-resistant depression; MADRS, Montgomery–Åsberg Depression Rating Scale; N, number; NS, not stated; PTSD, post-traumatic stress disorder.

**Table 2:** Adverse events of 5-MeO-DMT based on formulation.

AE	Formulation	
	Vaporized	Intranasal
Acute	Headache Head discomfort Nausea^1^ Sensory disturbance Hyperacusis Vision blurred Fatigue^1^ Feeling hot Clumsiness Anxiety Flashback* Abnormal dreams* Abdominal discomfort Muscle discomfort Muscle spasms Feeling abnormal Confusional state Euphoric mood Depressive symptom^1^ Hallucination Insomnia Mental fatigue Transient BP elevation^2^ Transient HR increase^1,2^	Nasal discomfort Nausea Vomitting Headache Administration site pain Chest discomfort Dizziness Pyrexia Gastroenteritis Back pain Hypoesthesia Limb discomfort Tremor Lacrimation increase Restlessness
SAE	NR	NR

* Might potentially refer to the phenomenon of ‘memory reactivation’ rather than flashbacks per se; ^1^moderate intensity; ^2^shortly after administration; AE, adverse event; BP, blood pressure; HR, heart rate; NR, not reported; SAE, serious adverse event; transient elevation of blood pressure and heart rate appears to be unrelated to the formulation and the inclusion in vaporized formulation only might mirror the differences in adverse events reporting.

**Table 3:** Comparison of vital signs at different time points after 5-MeO-DMT administration.

	Formulation						
	Vaporized			Intranasal			
	Baseline	1 h	3 h	Baseline	2 min	10 min	90 min
Systolic BP (mmHg; mean)	110 - 125	110 - 125	106 - 126	105 - 124	120 - 147	118 - 135	108 - 129
Diastolic BP (mmHg; mean)	71 - 79	68 - 77	67 - 77	NR			
HR (bpm; mean)	67 - 78	68 - 73	59 - 76	NR			

BP, blood pressure; bpm, beats per minute; HR, heart rate; NR, not reported.

Parameter score ranges are provided collectively for included doses.

#### 3.2.1 Vaporized formulation

The first study by Reckweg et al. (12) reported on the results from a phase 1 trial, which recruited 22 healthy volunteers with at least two previous psychedelic experiences and consisted of two single arms. The first arm employed a single-ascending dose regimen with dose levels of 5-MeO-DMT at 2, 6, 12, and 18 mg. The second arm evaluated individualized dose escalation (IDE) with doses of 6, 12, and 18 mg. The liquid product was administered following a standardized vaporization procedure. Adverse events (AEs) were recorded from the time of inclusion in the study until the study’s conclusion on day 7. Vital signs, including blood pressure (BP), heart rate (HR), oxygen saturation, body temperature, respiration rate, and electrocardiogram (ECG), were continuously monitored using a remote device. Blood and urine samples for laboratory safety analyses were collected at screening, at the end of the test day, and at the final visit. No AEs necessitated withdrawal from the study, and no serious adverse events (SAEs) were reported. Nearly all drug-related AEs were mild, with only two exceptions (“HR increased” and “fatigue”), which were of moderate intensity. Vital signs at 1 and 3 hours after administration were unaffected, and any AEs observed were mild and resolved spontaneously.

Another study conducted by Reckweg et al. (7) involved a phase 1 part examining two single dose levels (12 and 18 mg) with the primary endpoint focused on safety. The phase 2 part utilized an individualized dosing regimen (IDR) with up to three escalating doses (6, 12, and 18 mg) and the primary endpoint was efficacy defined as an overall score in Montgomery-Åsberg Depression Rating Scale (MADRS). Both phases involved 8 participants each, resulting in a final cohort of 16 patients. Eligible participants exhibited TRD, defined as the failure to respond to at least two adequate courses of pharmacological therapy or one adequate course of pharmacological therapy and at least one adequate course of evidence-based psychotherapy within the current episode. Those deemed eligible for the inclusion were required to taper-off antidepressant treatment and undergo a washout-procedure before entering the trial. Likewise, the drug was administered via inhalation, and safety protocols mirrored those outlined in the prior study. In this trial, no safety signals were detected regarding severe AEs, nor were any concerning findings observed in the safety laboratory analyses or psychiatric assessments.

#### 3.2.2 Intranasal formulation

A phase 1, double-blind, placebo-controlled trial enrolled 44 healthy, psychedelic-naive individuals to investigate the intranasal formulation of 5-MeO-DMT. Participants were stratified into 7 cohorts, with a maximum of 7 individuals per cohort. Within each cohort, 4 or 5 participants received a single intranasal dose (ranging from 1 to 12 mg), while the remaining individuals received a placebo. Safety and tolerability were continuously assessed throughout the trial, evaluating the incidence and severity of treatment-emergent adverse events (TEAEs), safety laboratory analyses, vital signs monitoring (including BP, HR, and temperature), ECGs, physical examinations, and suicide risk assessments. Ultimately, no SAEs were reported, nor any TEAE led to withdrawal from the study. Moreover, no clinically relevant results were observed in laboratory analyses, vital signs, ECGs, or physical examinations. Transient elevations in BP and heart rate resolved within a 90-minute timeframe. Importantly, no participants reported suicidal ideation or behavior, post-traumatic stress disorder symptoms, or hallucinogen-persisting perception disorder (13).

### 3.3 Risk of bias assessment

**Figure 2:**
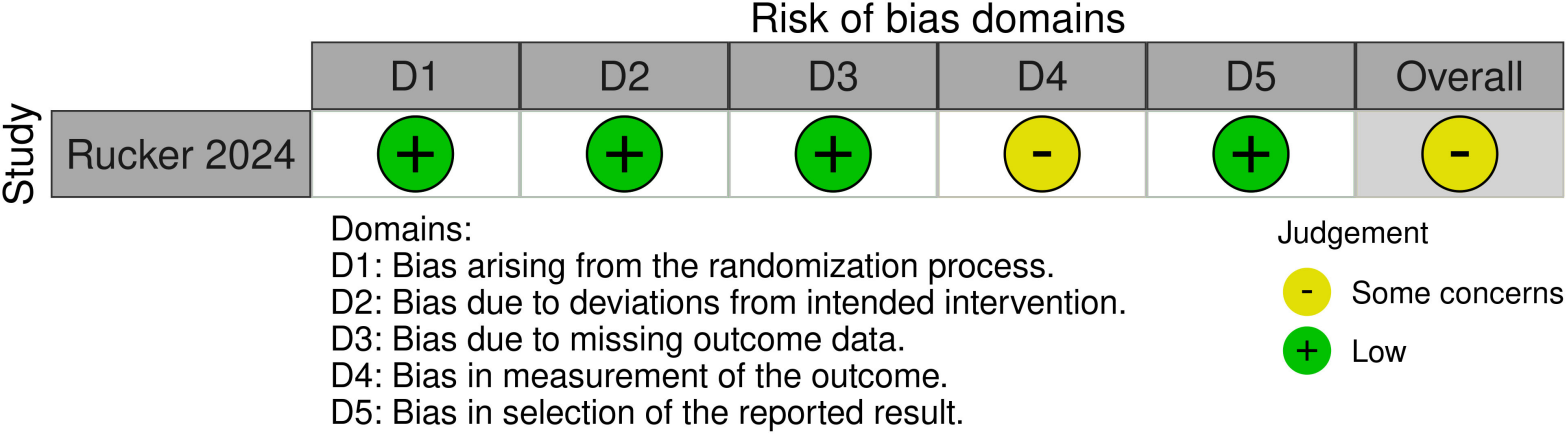
Risk of bias for randomized trials.

Newcastle-Ottawa Scale was employed to assess the quality of two non-randomized studies (7, 12) and were deemed to be of fair methodological quality. The third study (13) was evaluated using the RoB 2 tool for randomized controlled trials, resulting in a final assessment of ‘some concerns’. we. The quality of the included studies is depicted in **Table 4**, **Figure 2**.

**Table 4:** Risk of bias of non-randomized studies.

Study		Reckweg 2021 (12)	Reckweg 2023 (7)
**Selection**	Representativeness of the exposed cohort	0	0
	Selection of the non-exposed cohort	0	0
	Ascertainment of exposure	1	1
	Demonstration that outcome of interest was not presented at the start of the study	1	1
**Comparability**	Comparability of cohorts on the basis of the design or analysis controlled for confounders	1	1
**Outcome**	Assessment of outcome	1	1
	Was follow-up long enough for outcomes to occur	1	1
	Adequacy of follow-up of cohorts	1	1
**Total**		**6**	**6**

## 4 Discussion

This systematic review compiles and evaluates current literature of clinical trials on the safety and tolerability of 5-MeO-DMT administration in human subjects, confirming its favorable short-term safety and tolerability profile. The reported AEs are generally mild and resolve spontaneously. Importantly, no SAEs have been reported, and no AEs have resulted in patient withdrawal from the study. This is supportive of the evidence of the 5-MeO-DMT being the atypical psychedelic with a unique safety and tolerability profile. It supports the notion for the unmet clinical need for informative and conclusive safety and tolerability reporting in psychedelics where the class effect may not be applicable.

Regardless of the route of administration, both intranasal and vaporized formulations demonstrate comparable short-term safety and tolerability profiles for the doses studied in the trials (7, 12, 13). These findings suggest that the initial safety and tolerability of these formulations do not significantly differ based on how the drug is administered. Vital signs, particularly BP and HR, seem to be affected only briefly after administration. For instance, in the intranasal formulation, more patients experienced a pronounced increase in post-dose peak systolic BP than in placebo arm, yet all were clinically insignificant and were not classified as an AE. Similarly, patients exposed to the vaporized formulation also experienced an increase in BP. In these studies, this increase was classified as an AE, indicating inconsistencies in trial paradigms for AE reporting. Although single-ascending dosing and IDR protocols were employed in these studies, it remains premature to determine whether AEs are dose-dependent. Throughout the observation period, there were no instances of study participants opting to withdraw due to intolerance or AEs. This indicates a high level of tolerability and acceptability of 5-MeO-DMT among the study cohort.

Furthermore, no conclusions can be drawn on long-term safety or the evolution of AEs due to mere 7-day follow-up. For example, there are naturalistic reports (10, 19) on flashback experiences or ‘memory reactivation’, which are delayed in time, but due to the study design might not have been captured. Clinical trials often recruit highly selected patients under controlled conditions, which may not fully reflect the complexities and variability of real-world clinical needs. Complementing these trials with research in both non-clinical and naturalistic settings would provide more comprehensive and accurate picture of the true safety and tolerability profile of 5-MeO-DMT.

Adverse events induced in both healthy participants and patients are largely comparable across psychedelic compounds and are primarily influenced by whether the data were collected systematically or relied on spontaneous reports. Overall, the most commonly reported adverse events were anxiety, nausea, headache, and psychological or physical discomfort (20), which resembles the profile of 5-MeO-DMT. In contrast, there are reports on SAEs in psychedelic studies. During a psilocybin trial, a compound which is the most extensively studied psychedelic, Goodwin et al. (21) reported on a number of patients suffering from SAEs (including suicidal ideation). Some of them might potentially have been triggered by the initiation of antidepressant therapy or natural progression of the disease. Furthermore, there is a case that involved a patient with major depressive disorder who experienced bradycardia and hypotension following intravenous DMT administration (0.3 mg/kg bolus) (22). Another case involved patients administered 200 mg of LSD who developed acute anxiety and delusions, requiring treatment with lorazepam and (23). As for the impact of psychedelics on vital signs, LSD, psilocybin, and mescaline produced moderate elevations in blood pressure and heart rate. These physiological changes were dose-dependent and similar when comparing equivalent doses. On the other hand, DMT produces similar elevations when administered as an infusion, yet the increase is robust when administered as a bolus (20). For a detailed analysis of AEs in classic psychedelics readers are referred to another paper (24).

When establishing long term safety, addiction potential needs to be considered. Data from the Substance Abuse and Mental Health Services Administration, collected over the last 18 years from over 700,000 respondents, suggest that the lifetime use of 5-MeO-DMT or bufotenine/toad secretions is rare (25), and the addictive potential is rather low (26). In this aspect 5-MeO-DMT resembles classic psychedelics, for which modern research indicate rather low risk of dependence or compulsive use (24).

In the analyzed studies patients were free of antidepressant medication, thus it was not possible to observe potential drug interactions. Considering the metabolism of 5-MeO-DMT by monoamine oxidase A (MAO) and liver enzyme CYP2D6 (4). Since polymorphisms and variations in CYP2D6 activity can influence the metabolism of serotonin reuptake inhibitors (SSRI) (27), an interaction with MAO inhibitors (e.g., moclobemide) and serotonergic agents (e,g., SSRIs) can be suspected.

This paper has several limitations. Firstly, the small number of studies prevents us from drawing definitive conclusions. Taking this into account, future papers might as well incorporate real-world evidence on the subject to provide broader perspective on the matter. Secondly, only a total of 78 participants were recruited in included studies. Thirdly, subjects were followed up for a maximum of seven days, which limits our ability to assess long-term safety and tolerability. Fourthly, the inability of the placebos to replicate the effects of psychedelics compromises the blinding process. Fifthly, the definitions of treatment-emergent adverse events used in studies with regular antidepressants eg. SSRI might not be well suited for rapid-acting antidepressants (RAAD). With all limitations and cautions considered, the antidepressant effect of 5-MeO-DMT appears to be associated with the onset and intensity of a full-blown psychedelic experience (as defined by a peak or mystical experience). The occurrence of intensive peak experience could be considered an AE according to standard reporting criteria. However, these experiences might be better reconceptualized as a priming phenomenon necessary for the therapeutic response. This reconceptualization helps define the onset of the therapeutic effect and ensures that apparent lack of response is not mistakenly attributed to inadequate dosing. Thus, the conclusions drawn apply to 5-MeO-DMT only and shall not be generalized for all psychedelics.

There is no clear-cut definition for the safety and tolerability profile of psychedelics. These profiles may not be a class effect but rather specific to individual compounds. Therefore, adequate reporting of safety and tolerability is of paramount importance for research and development in both academia and industry, as well as for the regulatory process. Ensuring patient benefit and safety is essential, highlighting the translational importance of this information. The translational value of this observation lies in its potential to redefine both safety and efficacy reporting. Future studies should preferably be conducted in randomized, double-blind, placebo-controlled trials on larger samples with follow-up duration long enough to assess potential chronic AEs. To avoid selection bias, samples should include individuals with a similar history of psychedelic use.

## 5 Conclusion

In conclusion, this systematic review consolidates and assesses the existing literature from clinical trials regarding the safety and tolerability of 5-MeO-DMT administration in human subjects, affirming its favorable short-term safety and tolerability profile. Importantly, no SAEs have been documented, and no AEs led to participant withdrawal from the studies. These findings underscore the promising safety profile of 5-MeO-DMT in the context of human research trials. However, continued vigilance and further research are warranted to comprehensively understand its long-term safety implications.

## Data Availability

The original contributions presented in the study are included in the article/supplementary material, further inquiries can be directed to the corresponding author/s.
